# The latent profile structure of alexithymia in the elderly and its relationship to eating behaviors: the mediating role of physical activity

**DOI:** 10.3389/fpsyg.2025.1701168

**Published:** 2025-11-26

**Authors:** Bin Chen, Jing Yang, Wenying Huang, Wen Zhang, Chao Yang, Chang Hu

**Affiliations:** 1 Physical Education College, Jiangxi Normal University, Nanchang, China; 2 School of Psychology and Sociology, Mianyang Normal University, Mianyang, China

**Keywords:** physical activity, alexithymia, eating behaviors, Latent Profile Analysis, mediation analysis, older adults

## Abstract

**Objective:**

This study aimed to elucidate the psychological mechanisms underlying the relationship between alexithymia and problematic eating behaviors (EB) among older adults. Specifically, we examined whether physical activity (PA) mediated this association, and we further explored the heterogeneity of alexithymia using Latent Profile Analysis (LPA).

**Methods:**

A cross-sectional survey was conducted among 1,773 community-dwelling older adults in China. Participants completed validated questionnaires assessing alexithymia, PA, and EB. Mediation analysis tested the indirect effect of PA on the alexithymia-EB relationship, while LPA identified subgroups of individuals with distinct alexithymia profiles.

**Results:**

Mediation analysis revealed that PA significantly mediated the relationship between alexithymia and maladaptive EB, accounting for 18% of the total effect. LPA supported a three-profile solution: pervasive alexithymia (21.15%), adaptive (72.81%), and affective-cognitive dissociation (6.04%). Profile membership was differentially associated with health behaviors, with the pervasive group showing the most unfavorable outcomes (high EB, low PA), and the adaptive group demonstrating the most favorable pattern.

**Conclusion:**

These findings highlight PA as a key behavioral pathway through which alexithymia contributes to maladaptive eating in older adults. Moreover, alexithymia is not uniform but heterogeneous, with distinct profiles that confer varied health behavior risks. Interventions to improve eating habits in elderly populations may benefit from tailoring strategies to alexithymia subtypes and systematically promoting PA as an adaptive regulatory mechanism.

## 1 Introduction

Optimal nutrition is a cornerstone of lifelong health, profoundly influencing disease prevention, metabolic function, and overall vitality (Krasnow et al., 2014; Fadnes et al., 2022; Longo and Anderson, 2022). Eating behaviors (EB) represent the behavioral manifestations of dietary intake and are critical determinants of wellbeing (Dakanalis et al., 2023; Hill et al., 2021; Kärkkäinen et al., 2018; Huang et al., 2025a). As individuals transition into later life, the significance of these behaviors becomes even more pronounced (Kim et al., 2021; Walker-Clarke et al., 2022). The aging process is accompanied by unique physiological and psychosocial changes (Chen et al., 2025), a process that makes older adults particularly vulnerable, due to malnutrition risk, multimorbidity, and chronic diseases (Choi et al., 2019; Brownie, 2006; Dominguez et al., 2022). Consequently, maintaining adaptive eating patterns is not merely beneficial but essential for this demographic, serving as a primary defense against age-related morbidity, preserving functional autonomy, and supporting a higher quality of life (Hawash et al., 2023; Dent et al., 2023; Norman et al., 2021).

While the biochemical aspects of nutrition in aging are well-documented (Godos et al., 2020; Marx et al., 2020), the psychological factors that govern and often disrupt EB in the elderly remain less understood (Caso and Vecchio, 2022; Poggiogalle et al., 2020; Wang et al., 2023; Zhang et al., 2025). It is increasingly recognized that dietary choices are not made in a vacuum; they are deeply intertwined with an individual’s internal emotional and cognitive landscape (Spencer et al., 2017; Zheng et al., 2021). This recognition shifts the focus from simply prescribing dietary guidelines to understanding the underlying psychological mechanisms that enable or hinder their adoption (Wu et al., 2022; Biasini et al., 2021). A crucial, yet often overlooked, factor in this context is the personality trait of alexithymia.

Alexithymia, fundamentally characterized by a diminished capacity to identify, differentiate, and articulate one’s own emotional states, represents a significant challenge to self-regulation (Preece et al., 2021; Preece et al., 2023; Tella et al., 2020). Individuals with pronounced alexithymic traits struggle to process internal affective signals, which can lead to a reliance on external or somatic cues to interpret their inner world (Donges and Suslow, 2017; Lyvers et al., 2022; Preece and Gross, 2023). This deficit in emotional awareness can have far-reaching health implications (Wen et al., 2024; Neumann et al., 2025; Guidotti et al., 2025). Specifically, when faced with undifferentiated states of emotional distress, individuals may turn to maladaptive coping strategies, among which problematic eating is prominent (McAtamney et al., 2023; Rossi, 2025; Lanfredi et al., 2021). The act of eating can serve as a substitute behavior to soothe or distract from uncomfortable, yet poorly understood, internal turmoil (Bui et al., 2023). Given that alexithymia is prevalent in older age groups (Bos et al., 2022; Xu et al., 2024), it can be considered a critical variable in explaining a substantial proportion of the variance in their EB.

In addition, the pathway from alexithymia to problematic eating is unlikely to be direct and may be influenced by other health-related behaviors (Pink et al., 2019; Wheeler et al., 2005; Muir et al., 2024). Physical activity (PA), a behavior with well-established benefits for both emotional regulation and physical health, is a strong candidate as a mediating factor (Huang et al., 2025a; White et al., 2024). It is conceivable that the same emotional processing difficulties that contribute to alexithymia also reduce an individual’s motivation or ability to engage in purposeful PA (Proença Lopes et al., 2022; Shizuma et al., 2021; Hu et al., 2025), which itself is an adaptive coping mechanism (Perchtold-Stefan et al., 2020; Hu et al., 2025). This lack of engagement in PA could, in turn, heighten the reliance on maladaptive food-related coping (Qiu and Hou, 2020).

To further refine our understanding of this mechanism, it is crucial to recognize that alexithymia itself is not a monolithic construct (Ni and Fang, 2025; Bagby et al., 2021). Treating it as a single, uniform dimension may oversimplify. A “person-centered” methodology, such as Latent Profile Analysis (LPA), offers a more nuanced perspective by identifying distinct subgroups of individuals based on their specific patterns of alexithymic characteristics (Hong et al., 2020; Fernández et al., 2020; Spurk et al., 2020). This approach can reveal whether particular combinations of difficulties in identifying or describing feelings confer a differential risk for adverse health outcomes.

Therefore, this study aims to elucidate these complex relationships. Our primary objective is to investigate the mediating role of PA in the association between alexithymia and problematic EB among older adults. Secondly, we seek to apply LPA to uncover the potential heterogeneity of alexithymia in this population and to link these distinct profiles to health behavior outcomes. We hypothesize that PA is a key mechanism in this dynamic and that distinct, clinically meaningful profiles of alexithymia can be identified, each with a unique association with EB and PA levels.

## 2 Literature review and hypotheses development

### 2.1 Alexithymia and eating behavior

The conceptual link between alexithymia and problematic eating is primarily explained by the Emotion Regulation Model (Campos et al., 1989). This theoretical framework posits that individuals who lack the skills to effectively process and manage their emotions are more likely to engage in maladaptive behaviors to alleviate negative affective states (Preece et al., 2023; Casagrande et al., 2020). According to this model, eating—particularly of highly palatable foods—can function as a potent, albeit dysfunctional, tool for emotion regulation (Turton et al., 2017; Godet et al., 2022). For individuals with high alexithymic traits, internal states of anxiety, sadness, or anger are often experienced as confusing and overwhelming somatic sensations rather than distinct emotions (Quinto et al., 2025; Lundh and Simonsson-Sarnecki, 2001). Lacking the ability to identify the emotional source of their discomfort or to articulate their needs, they may misinterpret these internal cues as hunger or simply turn to eating as an accessible method to numb or distract from the distress (Goetz et al., 2020).

A substantial body of empirical research, though concentrated in younger adult and clinical samples, supports this connection. Studies have consistently demonstrated a positive correlation between higher levels of alexithymia and various forms of problematic eating, including binge eating, emotional eating, and restrictive behaviors (Yurtdaş Depboylu and Fındık, 2024; Favieri, 2021; Strodl and Wylie, 2020). This evidence suggests that the core deficits of alexithymia—difficulty identifying feelings (DIF) and difficulty describing feelings (DDF)—create a vulnerability to using food to manage emotional experiences (Obeid et al., 2021; Vuillier et al., 2020). However, this relationship has been significantly underexplored in the elderly. Older adults face a unique confluence of stressors, including chronic illness, bereavement, and social isolation, which can amplify emotional distress. It is therefore critical to examine whether the affect regulation model of eating behavior extends to this demographic, who may be particularly susceptible to the negative consequences of alexithymic traits.

### 2.2 The mediating role of physical activity

While the direct link is theoretically robust, the mechanisms that translate alexithymia into problematic eating behaviors warrant further investigation. One plausible intermediary is PA. PA is recognized not only for its physical health benefits but also as a powerful and adaptive strategy for emotion regulation (Teixeira et al., 2012; Zhang et al., 2024). Engagement in exercise has been shown to reduce symptoms of depression and anxiety, buffer the effects of stress, and enhance positive affect (Gerstberger et al., 2023; Bernstein and McNally, 2018). It provides a constructive outlet for personal emotions and can foster a sense of mastery and self-efficacy, thereby counteracting feelings of helplessness that may accompany emotional distress (Wu et al., 2022; Wang et al., 2020).

From this perspective, PA and problematic eating can be viewed as opposing coping strategies (Robinson et al., 2021; Varela et al., 2020). Individuals with effective emotion regulation skills are more likely to choose adaptive strategies, such as exercise, when faced with stress (Montana et al., 2020). Conversely, those with high alexithymia may be less likely to engage in PA for several reasons. First, their lack of emotional clarity may prevent them from recognizing the need for an emotional outlet (Gaggero et al., 2021). Second, an external orientation to thinking may lead them to devalue introspection-based activities or those requiring sustained internal motivation (Hill et al., 2024). This avoidance of an adaptive coping mechanism (PA) may leave a behavioral impact, increasing the likelihood of resorting to a maladaptive one (problematic eating) (Hao et al., 2022). Specifically, higher levels of alexithymia will be associated with lower levels of PA, which in turn will be associated with higher levels of problematic eating. This leads to our first hypothesis.

*Hypothesis 1 (H1):* PA will mediate the relationship between alexithymia and EB in older adults.

### 2.3 The profiles of alexithymia: a person-centered approach

While the variable-centered approach provides valuable insights into the general relationship between alexithymia and health behaviors, it inherently treats the construct as a monolithic entity. This assumption of homogeneity may mask crucial underlying complexities. In reality, the constituent features of alexithymia—namely, DIF, DDF, and externally oriented thinking (EOT)—may combine in systematically different ways across individuals, creating distinct, qualitatively different typologies of the trait (Fantini-Hauwel et al., 2025). Acknowledging this, recent research has increasingly adopted a person-centered methodology, such as LPA, to explore the latent structure of alexithymia (Hou et al., 2025; Alkan Härtwig et al., 2014).

This body of work has consistently revealed that alexithymia is indeed a heterogeneous construct. However, the exact number and nature of the identified profiles have varied across studies, likely due to differences in the populations and measurement tools used. For instance, research in samples of adolescents and young adults has yielded solutions ranging from three-profile models (typically a “low,” “moderate,” and “high” alexithymia group) (Alkan Härtwig et al., 2014) to more complex four-profile models that identify a distinct subgroup characterized by a specific deficit (Ni and Fang, 2025), such as pronounced difficulty in describing feelings alongside relatively intact emotional identification. These findings underscore the importance of not just asking “how much” alexithymia an individual exhibits, but rather “what kind” of alexithymic profile they present.

Critically, a significant gap exists in this literature: to date, this person-centered exploration of alexithymia has not been extended to an elderly population. This omission is particularly striking, given that older adults may experience unique emotional and cognitive changes that could shape the manifestation of alexithymic traits differently than in younger cohorts. Therefore, applying LPA to this demographic is not merely a replication but a crucial step toward understanding whether the established profile structures are universal or age-specific. This exploration leads to our second hypothesis, which is foundational to the subsequent behavioral analysis:

*Hypothesis 2 (H2):* It is hypothesized that an LPA will uncover a distinct and meaningful latent profile structure of alexithymia within a sample of older adults.

The identification of such profiles holds significant clinical and theoretical implications, as it enables a more nuanced investigation of risk. If distinct subgroups exist, they are likely to be differentially associated with health outcomes. A profile defined by globally high deficits across all dimensions of alexithymia would logically be expected to confer the highest risk for maladaptive coping. Conversely, other profiles may reveal which specific aspect of alexithymia (e.g., the cognitive difficulty in identifying feelings versus the behavioral difficulty in expressing them) is the primary driver of engagement in health-detrimental behaviors, such as physical inactivity and problematic eating. This provides a more nuanced framework than a simple total score and leads to our final, consequential hypothesis:

*Hypothesis 3 (H3):* Different alexithymia profiles will be significantly associated with both PA levels and EB.

### 2.4 The present study

In summary, the present research has two primary aims. The first aim is to examine the mediating effect of PA on the relationship between overall alexithymia and problematic EB, thereby testing the proposed affect regulation pathway (H1). The second, and more exploratory, aim is to utilize LPA to first identify the latent profile structure of alexithymia in the elderly (H2), and subsequently to investigate how membership in these empirically derived profiles is associated with differential levels of PA and problematic eating (H3). By pursuing these dual objectives, this study aims to provide a more comprehensive and nuanced understanding of the psychological mechanisms that influence key health behaviors among the growing population of older adults.

## 3 Materials and methods

### 3.1 Participants and procedure

This study employed a cross-sectional design to investigate the relationship between alexithymia, PA, and EB among older adults. The sample size was determined *a priori* using G*Power 3.1 software. For a simple mediation model, with a medium effect size (*f*^2^ = 0.15), a significance level (*α*) of 0.05, and a desired statistical power of 95%, the analysis indicated that a minimum of 107 participants were required. The final sample size of 1,773 exceeded the minimum by a considerable margin, ensuring sufficient statistical power to detect the hypothesized effects and enhancing the reliability of the findings.

Participants were recruited from May to June 2025 across three provinces in China: Jiangxi, Hunan, and Yunnan. The inclusion and exclusion criteria were established to ensure the homogeneity of the Sample and the validity of the data. Inclusion criteria: (1) Aged 60 years or older. (2) Able to provide informed consent. (3) Fluent in Mandarin and able to comprehend the questionnaire. (4) Willing to participate voluntarily in the study. Exclusion criteria: (1) Diagnosis of a severe psychiatric disorder (e.g., schizophrenia, bipolar disorder). (2) Presence of a neurocognitive disorder (e.g., dementia, Alzheimer’s disease) that would impair their ability to complete the questionnaire accurately. (3) A physical disability that would prevent them from engaging in PA. (4) Currently undergoing treatment for problematic eating.

A multi-stage random sampling was employed to recruit a diverse and representative sample of older adults. First, several cities were randomly selected from each of the three provinces. Next, within these cities, residential communities and public parks were chosen as recruitment sites. Data collection was conducted through an offline, paper-and-pencil survey. Trained research assistants approached potential participants, explained the purpose and procedure of the study, and obtained written informed consent. Participants were assured of the confidentiality and anonymity of their responses. Each participant received a compensation of 5 RMB for their contribution. Initially, 1,926 questionnaires were distributed. All returned questionnaires were meticulously screened for missing data and outliers; any instances of missing data or outliers were excluded from the analysis to ensure the quality of the dataset. After a thorough review, 153 questionnaires were excluded due to incomplete data or failure to meet the inclusion criteria. This resulted in a final sample of 1,773 valid questionnaires for data analysis, yielding a response rate of 92.0%. The final dataset used for analysis contained no missing data or abnormal data points. The age of the Sample ranged from 60 to 83 (*M* = 67.90, *SD* = 5.43). The Institutional Ethics Review Board at our institution has approved this study (IEB-JXNU-PEC-2025014).

### 3.2 Measure

#### 3.2.1 Alexithymia

Alexithymia was measured with the 20-item Toronto Alexithymia Scale (TAS-20), a widely used instrument developed by Bagby et al. (1994). This self-report questionnaire evaluates three core facets of the alexithymia construct: DIF, DDF, and EOT. Respondents rated their agreement with each item (e.g., “I’d rather talk to people about their daily activities than their inner feelings”) on a five-point Likert scale, anchored by 1 (“completely disagree”) and 5 (“completely agree”). For this study, all reverse-scored items were converted to forward scoring, and the mean score across all items was used for analysis. A higher average score reflects a greater severity of alexithymic traits. The scale demonstrated strong internal consistency within the current Sample, achieving a Cronbach’s alpha of 0.902.

#### 3.2.2 Eating behaviors

Eating behavior (EB) was evaluated using the 7-item short form of the Sakata Eating Behavior Scale. This instrument was originally developed by Tayama et al. (2017) and subsequently validated for use within a Chinese population by Ge et al. (2023). Participants rated their responses on a four-point Likert scale for each of the seven items (e.g., “When buying food, I am not content unless I buy more than necessary”). For this analysis, the average score was calculated, with a higher mean indicating a stronger inclination toward problematic EB. The scale exhibited high internal consistency in the current investigation, achieving a Cronbach’s alpha of 0.763.

#### 3.2.3 Physical activity

To assess PA, this study utilized the Physical Activity Rating Scale (PARS), an instrument adapted by Liang (1994). The scale evaluates three key dimensions of PA: intensity, duration, and frequency, with each dimension rated on a 5-level scale. The reliability of this measure has been previously established within Chinese populations by researchers such as Yang et al. (2024) and Li et al. (2023). For this investigation, we calculated the mean score across the three dimensions. Consequently, a higher average score is indicative of a greater level of PA engagement. The scale demonstrated good internal consistency in the present study, with a Cronbach’s alpha of 0.731.

#### 3.2.4 Covariates

Based on a review of prior research, several demographic variables were selected and included in the analysis as covariates to control for their potential confounding effects on EB and PA. These variables, controlled for in the regression models, included age (Wang et al., 2023) (treated as a continuous variable), gender (Dubois et al., 2022) (categorized as male or female), education (Simone et al., 2022) (categorized as primary school and below, junior high school, high school, or bachelor’s degree and higher), place of birth (Kvalsvik et al., 2021) (dichotomized as city or rural), and ethnicity (Teh et al., 2023) (dichotomized as Han or minority). Furthermore, Health Status (da Silva et al., 2022) and Healthcare expenditures (Caso and Vecchio, 2022) were also considered for inclusion. However, T-test analyses indicated that these two variables were not significantly associated with the primary variables of interest in this study. Thus, they were excluded from the final model.

### 3.3 Data analysis

All statistical analyses were conducted using SPSS v.26.0 and Mplus v.8.3, with statistical significance set at *p* < 0.05. The analysis began with preliminary data screening, including the computation of descriptive statistics and bivariate correlations to characterize the Sample and examine initial variable relationships. We assessed univariate normality via skewness and kurtosis. All variables met commonly recommended thresholds for approximate normality (|skewness| < 2, |kurtosis| < 8) (Hu et al., 2025), supporting the use of parametric tests. To ensure the integrity of subsequent regression analyses, a multicollinearity diagnostic was performed. The Variance Inflation Factor (VIF) scores confirmed that the assumption of no multicollinearity was met. The central hypothesis was tested using a mediation analysis with the PROCESS macro (Model 4) in SPSS. This assessed whether PA mediated the link between alexithymia and problematic EB. The significance of the indirect effect was determined using 5,000 bootstrap resamples. Subsequently, to investigate the heterogeneity of alexithymia, LPA was employed to identify distinct subgroups within the Sample. The optimal number of profiles was determined by comparing model fit indices (e.g., AIC, BIC, ABIC) and the Lo-Mendell-Rubin Likelihood Ratio Test (LMR-LRT). Finally, a one-way analysis of variance (ANOVA) was conducted to compare the identified alexithymia profiles on the outcome variables of EB and PA. Significant main effects were followed up with Bonferroni-corrected post-hoc tests to ascertain specific pairwise differences between the latent profiles.

## 4 Results

### 4.1 Demographic information of older adults

Group comparisons identified several significant differences. For EB, women reported more problematic patterns than men (*p* < 0.001). Education was also related to EB (*p* < 0.01), with higher education being associated with fewer problematic behaviors. Participants born in urban areas showed higher eating behavior scores than those born in rural areas (*p* < 0.001). Likewise, minority participants demonstrated higher scores on both alexithymia and EB compared with Han participants (*p* < 0.001) (Table 1).

**Table 1:** Demographic characteristics and group differences among older adults.

	*n*	Proportion	Alexithymia	Eating behaviors	Physical activity
Gender			−0.711	−7.444^***^	2.298^*^
Men	862	48.60%			
Female	911	51.40%			
Education			2.602	4.787^**^	1.346
Primary school and below	776	43.80%			
Junior high school	802	45.20%			
High School	159	9.00%			
Bachelor’s degree or higher	36	2.00%			
Place of birth			0.982	6.173^***^	−0.757
City	921	51.90%			
Rural	852	48.10%			
Ethnicity			−4.695^***^	4.783^***^	−1.390
Han	1,557	87.80%			
Minority	216	12.20%			
Marital status			0.963	2.370	0.550
Married	1,087	61.30%			
Divorce	224	12.60%			
Unmarried	36	2.00%			
Widowed	426	24.00%			
Health status			−0.560	−0.584	0.447
Independent	1,555	87.70%			
Dependent	218	12.30%			
Healthcare expenditures			−1.524	−0.172	1.268
Self-founded	217	12.20%			
Health Insurance	1,556	87.80%			

Unpaired t-test and ANOVA results (two-tailed test). ^*^p < 0.05,^**^p < 0.01,^***^p < 0.001. Significance levels apply to subsequent tables unless otherwise noted.

### 4.2 Correlation analysis

Descriptive information and bivariate associations are reported in Table 2. The distributional plausibility of the variables was ascertained through an examination of skewness and kurtosis. The results indicated that the variables exhibited skewness values (|skew| ≤ 0.50) and kurtosis values (|kurtosis| ≤ 0.75), thereby substantiating the validity of parametric and bootstrap inferences. Alexithymia showed a positive association with problematic EB (*r* = 0.257, *p* < 0.01) and a negative association with PA (*r* = −0.299, *p* < 0.01). PA was inversely related to problematic EB (*r* = −0.240, *p* < 0.01). These correlations support examining PA as a potential pathway linking alexithymia with eating outcomes.

**Table 2:** Variable statistics and relationships in alexithymia, physical activity, and eating behavior.

	*M*	*SD*	Skewness	Kurtosis	Alexithymia	EB	PA
Alexithymia	2.714	0.724	0.494	0.228	1		
EB	2.379	0.650	0.038	−0.741	0.257^**^	1	
PA	2.592	0.650	0.371	−0.640	−0.299^**^	−0.240^**^	1

Pearson correlations (two-tailed).**p < 0.01.

### 4.3 The mediation analyses

Before testing the mediation model, multicollinearity was examined. The variance inflation factor (VIF = 1.098) indicated no concerns, thus meeting the assumptions for regression analysis.

**Figure 1:**
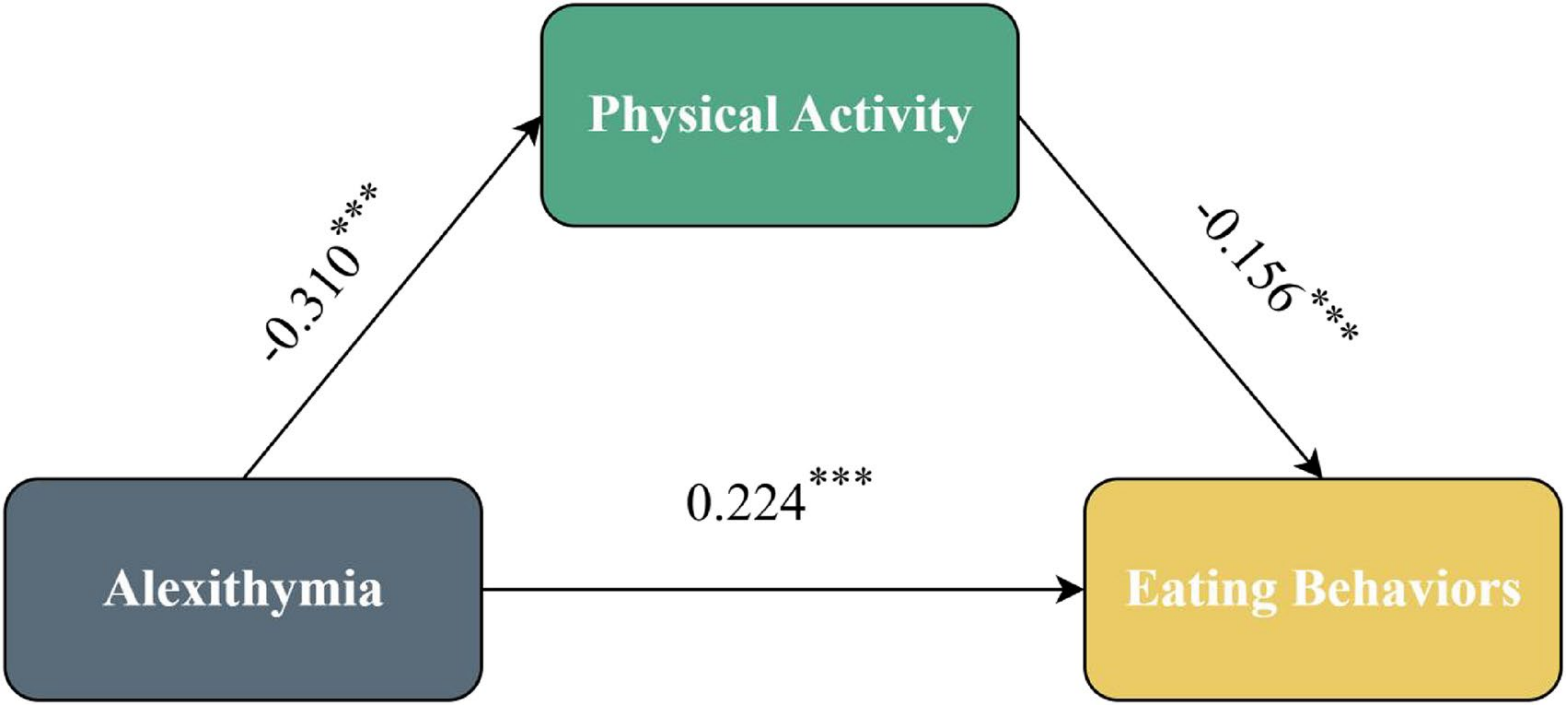
Mediation effect diagram.

The results of the mediation analysis, incorporating covariates, are summarized in Table 3. Alexithymia was significantly associated with PA (*β* = −0.310, *t* = −13.576, *p* < 0.001) and with EB (*β* = 0.224, *t* = 9.621, *p* < 0.001). PA was also significantly related to EB (*β* = −0.156, *t* = −6.758, *p* < 0.001) (Figure 1).

**Table 3:** Mediation effect regression results.

Variables	Model1		Model2		Model3	
	(PA)		(EB)		(EB)	
	*β*	*t*	*β*	*t*	*β*	*t*
Alexithymia	−0.310	−13.576	0.224	9.621	0.272	12.141
PA			−0.156	−6.758		
Ethnicity	0.244	3.491	−0.394	−5.795	−0.432	−6.297
Place of birth	0.010	0.210	−0.248	−5.635	−0.249	−5.598
Gender	−0.089	−1.957	0.290	6.583	0.304	6.819
Education	0.062	1.963	−0.058	−1.882	−0.067	−2.173
Age	0.001	0.177	0.002	0.387	0.001	0.354
*R* ^2^	0.100		0.155		0.133	
*F*	32.802^***^		46.206^***^		45.152^***^	

Multiple linear regression (PROCESS Model 4 framework).***p < 0.001.

Bootstrap testing with 5,000 resamples confirmed a significant indirect pathway of alexithymia on EB through PA (indirect effect = 0.048, 95% CI [0.033, 0.065]), accounting for 18% of the total effect (Table 4). The direct pathway remained significant (effect = 0.224, 95% CI [0.178, 0.269]), accounting for 82% of the total effect. These results suggest that PA partially explains the association between alexithymia and problematic EB.

**Table 4:** Mediating effects of physical activity between alexithymia and eating behavior.

	Effect	Boost se	95%CI		Relativistic effect
			LLCI	ULCI	
Total	0.272	0.022	0.228	0.316	
Alexithymia → EB	0.224	0.023	0.178	0.269	82%
Alexithymia → PA → EB	0.048	0.008	0.033	0.065	18%

Bootstrapped indirect effects with 5,000 resamples; bias-corrected 95% CI.

### 4.4 Latent profile analysis

**Figure 2:**
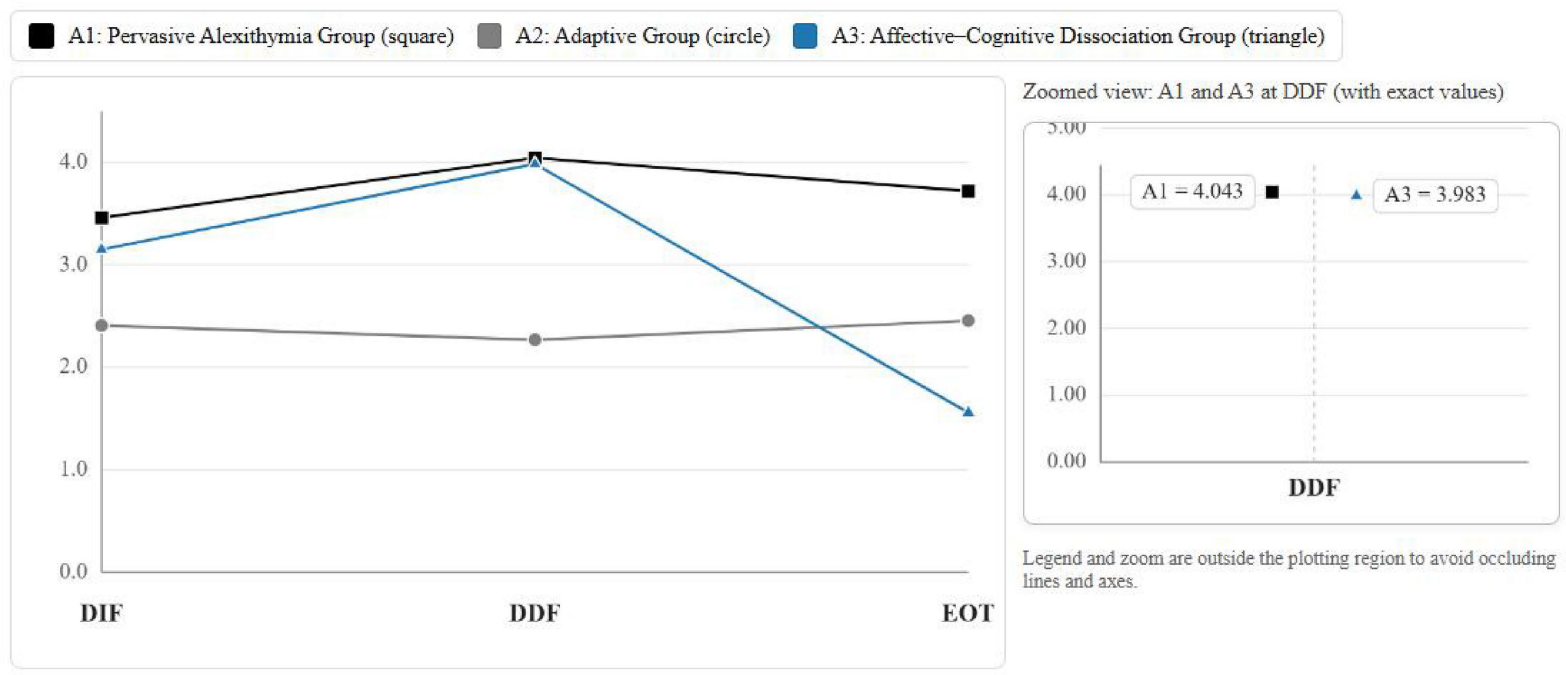
Latent profiles of alexithymia.

LPA models from one to five classes were compared (Table 5). The three-class solution showed the most favorable balance of fit indices (e.g., lower AIC/BIC values), a significant LMR-LRT and BLRT, and a high entropy value (0.891), indicating adequate classification precision. On this basis, the three-class solution was selected. Based on the mean scores across each dimension of the latent alexithymia profiles (Figure 2), they are sequentially named as follows: Class A1: Pervasive Alexithymia Group (*n* = 375, 21.15%), Class A2 Adaptive Group: (*n* = 1,291, 72.81%), Class A3 Affective-Cognitive Dissociation Group (*n* = 107, 6.04%).

**Table 5:** Latent Profile Analysis models.

Model	AIC	BIC	aBIC	LMR(P)	BLRT(P)	Entropy	Categorical probability%
Class 1	14,010.515	14,043.397	14,024.336				
Class 2	13,136.176	13,190.980	13,159.211	<0.0001	<0.0001	0.838	80.65%/19.35%
Class 3	12,911.923	12,988.649	12,944.172	<0.0001	<0.0001	0.891	21.15%/72.81%/6.04%
Class 4	12,651.896	12,750.544	12,693.359	<0.0001	<0.0001	0.833	57.81%/13.54%/8.12%/20.53%
Class 5	12,427.042	12,547.612	12,477.719	<0.0001	<0.0001	0.856	13.71%/8.23%/48.05%/7.90%/22.11%

Latent Profile Analysis.

### 4.5 Effect of latent profile classification

Analysis of variance revealed significant differences between the three profiles for both PA (*F* = 31.411, *p* < 0.001) and EB (*F* = 23.173, *p* < 0.001) (Table 6).

**Table 6:** Group differences across latent profiles.

Variable	A1 (375)	A2 (1,291)	A3 (107)	*η* ^2^	*F*
PA	2.249 ± 1.0749	2.691 ± 0.881	2.601 ± 1.245	0.034	31.411^***^
EB	2.512 ± 0.74859	2.369 ± 0.618	2.040 ± 0.511	0.026	23.173^***^

One-way ANOVA result (two-tailed), A1 (Pervasive Alexithymia Group), A2 (Adaptive group), A3 (Affective–Cognitive Dissociation group).***p < 0.001.

*Post hoc* Bonferroni comparisons (Table 7) showed that EB followed the gradient A1 > A2 > A3, with the Pervasive Alexithymia group demonstrating the most problematic EB, the Adaptive group showing intermediate levels, and the Affective–Cognitive Dissociation group the lowest. For PA, the order was A2 > A3 > A1. The Adaptive group reported the highest PA, followed by the Affective–Cognitive Dissociation group, with the Pervasive Alexithymia group lowest.

**Table 7:** Multiple comparisons.

Bonferroni	(I) Class	(J) Class	Mean difference (I-J)	STD. Error	Sig.	95% CI		Outcome
Dependent variable						Lower bound	Upper bound	
EB	A1	A2	0.144^*^	0.038	<0.001	0.053	0.234	
		A3	0.472^*^	0.070	<0.001	0.304	0.641	
	A2	A1	−0.144^*^	0.038	<0.001	−0.234	−0.053	
		A3	0.329^*^	0.065	<0.001	0.174	0.483	
	A3	A1	−0.472^*^	0.070	<0.001	−0.641	−0.304	
		A2	−0.329^*^	0.065	<0.001	−0.483	−0.174	A1 > A2 > A3
PA	A1	A2	−0.442^*^	0.038	<0.001	−0.575	−0.308	
		A3	−0.351^*^	0.056	<0.01	−0.602	−0.103	
	A2	A1	0.442^*^	0.104	<0.001	0.308	0.575	
		A3	0.089^*^	0.056	>0.05	−0.140	0.318	
	A3	A1	0.353^*^	0.096	<0.01	0.103	0.602	
		A2	−0.089^*^	0.104	>0.05	−0.318	0.140	A1 < A3 < A2

Bonferroni-corrected post-hoc pairwise comparisons.*p < 0.05.

Taken together, these findings support the heterogeneity of alexithymia and indicate that subgroup membership is differentially associated with health-related behaviors.

## 5 Discussion

### 5.1 The mediating role of PA

The present findings provide robust evidence that PA partially mediates the relationship between alexithymia and problematic EB in older adults. Mediation analysis revealed that PA accounted for 18% of the total effect, indicating that reduced engagement in adaptive health behaviors makes a meaningful contribution to the influence of alexithymia on dietary regulation.

These results align with prior empirical studies suggesting that alexithymia is often accompanied by difficulties in adopting and maintaining health-promoting behaviors, including exercise (Proença Lopes et al., 2022; Shizuma et al., 2021). Moreover, a growing body of research has demonstrated the protective influence of PA on emotional health and EB. For example, exercise has been shown to buffer the effect of stress and improve emotion regulation skills (Gerstberger et al., 2023; Bernstein and McNally, 2018; Huang et al., 2025b), while insufficient PA is concurrently linked to heightened risk of problematic eating patterns (Robinson et al., 2021; Hao et al., 2022). The present study extends this literature by confirming a mediational pathway within an elderly population, a demographic that has been underrepresented in previous research.

Theoretically, these findings can be understood through the lens of the dual pathway model of coping (Keech and Hamilton, 2022). On one side, alexithymic individuals often lack the capacity to identify and manage internal emotional cues, leaving them vulnerable to maladaptive behaviors such as emotional or excessive eating (Casagrande et al., 2020). On the other hand, PA represents a constructive outlet for releasing emotional tension, enhancing self-efficacy, and reinforcing adaptive regulatory strategies (Perchtold-Stefan et al., 2020; Wu et al., 2022). When alexithymic traits diminish the likelihood of engaging in PA, this adaptive route is blocked, thereby amplifying reliance on less constructive compensatory behaviors (Luminet and Nielson, 2025).

From a practical perspective, the identification of PA as a mediator highlights a promising target for intervention. Programs designed to mitigate problematic eating in older adults with alexithymic tendencies should not focus solely on dietary education. Still, they should also incorporate components that foster engagement in enjoyable and sustainable physical activities. Clinicians and community health practitioners might therefore consider integrating structured PA programs as part of broader psychosocial interventions to enhance emotion regulation and reduce maladaptive eating.

### 5.2 Latent profile of alexithymia

Our LPA revealed a three-class solution, confirming that alexithymia in older adults is heterogeneous rather than uniform. This pattern parallels person-centered studies in younger populations (Bagby et al., 2021; Alkan Härtwig et al., 2014), while extending the evidence into later life. Conceptually, the findings reinforce alexithymia as a multidimensional construct with subgroups reflecting distinct combinations of emotional processing difficulties.

The first class, the Pervasive Alexithymia group (21.15%), presented uniformly high scores across all dimensions of alexithymia. The emergence of this profile may be related to cumulative effects of chronic health problems, social losses, and reduced opportunities for emotional communication in late life (Levinsky and Schiff, 2021; Rook and Charles, 2017). For some individuals, lifelong difficulties in recognizing emotions may be exacerbated by the stress of widowhood or diminished peer networks, resulting in consistently severe deficits across domains (Antonucci et al., 2019).

The second and largest class, the Adaptive group (72.81%) reported low scores across all dimensions, reflecting relatively intact emotional processing. This sizable subgroup may comprise individuals exposed to protective factors such as higher educational attainment, richer social participation (e.g., community clubs, volunteer work), and sustained engagement in cognitively stimulating activities, all of which are known to support emotional awareness in later life (Cabello et al., 2014; Andonian and MacRae, 2011).

Finally, the Affective-Cognitive Dissociation group (6.04%), exhibited moderate alexithymia, characterized by difficulties in identifying and describing feelings, while scoring lower on externally oriented thinking. Generational and cultural influences might explain this profile: many older Chinese adults grew up in contexts where direct discussion of emotions was discouraged, which can foster difficulties in verbalizing affect even if other cognitive capacities remain relatively intact (Lui, 2015).

Altogether, these findings suggest that alexithymia patterns in older adults emerge from the interplay of both life-course exposures and aging-specific stressors, providing a foundation for designing interventions responsive to subgroup-specific contexts.

### 5.3 Effect of latent profile classification

Comparisons across the three latent profiles revealed significant group differences in both PA and EB. The Pervasive Alexithymia group consistently showed the most maladaptive outcomes. Members of this group reported the highest scores for problematic eating and the lowest levels of PA. Thereby, this pattern aligns with prior evidence that severe and global deficits in emotional processing leave individuals with few constructive coping routes, heightening reliance on maladaptive strategies such as problematic eating (McAtamney et al., 2023; Strodl and Wylie, 2020).

By contrast, the Affective-Cognitive Dissociation group demonstrated an intriguing combination of moderately elevated PA and the lowest problematic EB levels. Despite their difficulties in identifying and describing feelings, this group maintained relatively lower externally oriented thinking, possibly preserving some behavioral flexibility. Prior research has highlighted that individuals with partial alexithymic deficits may compensate for poor emotional awareness by engaging in structured behavioral outlets, such as exercise (Hu et al., 2025; Perchtold-Stefan et al., 2020). For these older adults, PA likely functions as a pragmatic, nonverbal regulatory route that is readily accessible despite limited emotional clarity, helping to mitigate maladaptive eating and supporting higher activity engagement relative to more globally impaired profiles.

Finally, the Adaptive group reported low levels of problematic eating and the highest PA among the three profiles. In contrast to the Affective-Cognitive Dissociation group, their relatively intact emotional processing likely enables a broader repertoire of regulatory strategies (e.g., emotional articulation, cognitive reappraisal, and social coping), reducing reliance on any single outlet. This balanced profile suggests that diverse regulatory resources—combined with high PA—offer a particularly protective framework against maladaptive eating and behavioral inflexibility.

Theoretically, these findings underscore that not all alexithymia-related risks stem solely from severity; profile-specific configurations also matter. Practically, interventions should differentiate approaches: Pervasive group members may need comprehensive programs integrating emotion regulation training and lifestyle modification; Dissociation group individuals could benefit from reinforcing structured PA while cultivating emotional skills; and Adaptive individuals may be best supported through maintenance of diverse coping resources. Such tailored strategies promise more effective and resource-efficient interventions in promoting healthy aging.

### 5.4 Strengths and limitations

This study has several notable strengths. First, it employed a large and diverse sample of 1,773 community-dwelling older adults across multiple provinces in China, substantially exceeding the minimum power requirement and enhancing the generalizability of findings. Second, the dual analytical approach—combining mediation modeling with LPA—allowed us not only to test mechanistic pathways linking alexithymia with maladaptive eating but also to uncover heterogeneity within the construct. This person-centered approach provides a more nuanced understanding than variable-centered methods alone. Third, validated and widely used instruments (e.g., TAS-20, Sakata Eating Behavior Scale, PARS) were applied, with good internal consistency in the present Sample, ensuring measurement reliability.

Despite these strengths, several limitations should be acknowledged. The cross-sectional design precludes causal inference; longitudinal or experimental studies are required to confirm whether alexithymia leads to problematic eating through reduced PA over time. In addition, all measures were based on self-report questionnaires, which may be subject to recall bias and social desirability effects. Incorporating objective measures—such as accelerometers for PA or ecological momentary assessment for EB—could provide more robust evidence. Furthermore, while the large Sample was diverse in regional representation, it was limited to Chinese older adults; cultural factors around emotion expression and health behavior may influence both the prevalence of alexithymia profiles and their behavioral correlates. Replication in other cultural contexts is needed. Moreover, despite the high response rate, generalizability beyond the sampled provinces still warrants caution. In addition, We acknowledge that coding ethnicity as Han versus minority, while common and practical, may obscure heterogeneity among minority groups; future studies should oversample specific minority populations to enable more granular analyses. Finally, unmeasured variables such as personality traits, stress exposure, and social support networks may further account for variability in the observed relationships and warrant exploration in future research.

Taken together, these limitations suggest that while the present findings advance our understanding of alexithymia and health behaviors in older adults, they should be interpreted cautiously. Future longitudinal and cross-cultural studies are required to strengthen causal claims and broaden the applicability of these findings.

## 6 Conclusion

The present study provides new evidence regarding how alexithymia influences problematic EB in late life. First, PA was identified as a significant mediator, demonstrating that reduced engagement in PA partially explains the pathway from emotional processing deficits to maladaptive eating. Second, LPA revealed that alexithymia among older adults is not uniform but heterogeneous, with distinct subgroups differing in emotional characteristics and associated health behaviors. These findings emphasize that both the quantity and the configuration of alexithymic traits shape older adults’ coping patterns.

In conclusion, by addressing both common mechanisms and latent profiles, this study advances knowledge on the links between alexithymia, PA, and EB in older adults. Future longitudinal and cross-cultural research is encouraged to validate these findings and inform the development of targeted, profile-based interventions that promote healthier aging.

## Data Availability

The original contributions presented in the study are included in the article/supplementary material, further inquiries can be directed to the corresponding authors.

## References

[ref1] Alkan Härtwig E. Crayen C. Heuser I. Eid M. (2014). It’s in the mix: psychological distress differs between combinations of alexithymic facets. Front. Psychol. 5:1259. doi: 10.3389/fpsyg.2014.01259, PMID: 25429275 PMC4228974

[ref2] Andonian L. MacRae A. (2011). Well older adults within an urban context: strategies to create and maintain social participation. Br. J. Occup. Ther. 1, 2–11. doi: 10.4276/030802211X12947686093486

[ref3] Antonucci T. C. Ajrouch K. J. Webster N. J. Zahodne L. B. (2019). Social relations across the life span: scientific advances, emerging issues, and future challenges. Annu. Rev. Dev. Psychol. 1, 313–336. doi: 10.1146/annurev-devpsych-121318-085212

[ref4] Bagby R. M. Parker J. D. A. Taylor G. J. (1994). The twenty-item Toronto alexithymia scale—I. Item selection and cross-validation of the factor structure. J. Psychosom. Res. 38, 23–32. doi: 10.1016/0022-3999(94)90005-1, PMID: 8126686

[ref5] Bagby R. M. Sanches M. Carnovale M. Taylor G. J. (2021). An evaluation of alexithymia subtypes using latent profile analysis. Psychiatry Res. 299:113840. doi: 10.1016/j.psychres.2021.113840, PMID: 33667948

[ref6] Bernstein E. E. McNally R. J. (2018). Exercise as a buffer against difficulties with emotion regulation: a pathway to emotional wellbeing. Behav. Res. Ther. 109, 29–36. doi: 10.1016/j.brat.2018.07.010, PMID: 30081242

[ref7] Biasini B. Rosi A. Giopp F. Turgut R. Scazzina F. Menozzi D. (2021). Understanding, promoting and predicting sustainable diets: a systematic review. Trends Food Sci. Technol. 111, 191–207. doi: 10.1016/J.TIFS.2021.02.062

[ref8] Bos P. Voshaar R. C. O. Hanssen D. J. C. (2022). Prevalence and correlates of alexithymia in older persons with medically (un)explained physical symptoms. Int. J. Geriatr. Psychiatry 37, 1–11. doi: 10.1002/gps.5736, PMID: 35584287 PMC9325002

[ref9] Brownie S. (2006). Why are elderly individuals at risk of nutritional deficiency? Int. J. Nurs. Pract. 12, 110–118. doi: 10.1111/J.1440-172X.2006.00557.X, PMID: 16529597

[ref10] Bui M. Krishen A. Kemp E. (2023). It’s a force of habit: influences of emotional eating on indulgent tendencies. J. Consum. Mark. 40, 445–457. doi: 10.1108/JCM-01-2022-5146

[ref11] Cabello R. Navarro B. Latorre J. M. Fernández-Berrocal P. (2014). Ability of university-level education to prevent age-related decline in emotional intelligence. Front. Aging Neurosci. 6:37. doi: 10.3389/fnagi.2014.00037 24653697 PMC3949193

[ref12] Campos J. J. Campos R. G. Barrett K. C. (1989). Emergent themes in the study of emotional development and emotion regulation. Dev. Psychol. 25, 394–402. doi: 10.1037/0012-1649.25.3.394

[ref13] Casagrande M. Boncompagni I. Forte G. Guarino A. Favieri F. (2020). Emotion and overeating behavior: effects of alexithymia and emotional regulation on overweight and obesity. Eat. Weight Disord. 25, 1333–1345. doi: 10.1007/s40519-019-00767-9, PMID: 31473988

[ref14] Caso G. Vecchio R. (2022). Factors influencing independent older adults (un)healthy food choices: a systematic review and research agenda. Food Res. Int. 158:111476. doi: 10.1016/j.foodres.2022.111476, PMID: 35840197

[ref15] Chen B. Huang W. Hu C. (2025). The relationship between positive exercise experiences and mobile phone addiction tendencies in older adults: a cross-lagged study. Front. Public Health 13:1710048. doi: 10.3389/fpubh.2025.1710048 41323583 PMC12661996

[ref16] Choi Y. J. Ailshire J. A. Crimmins E. (2019). Dietary intake and nutritional risk among older Americans. Innov. Aging 3:939. doi: 10.1093/geroni/igz038.3414, PMID: 40799322

[ref17] da Silva L. S. L. de Freitas Batalhão D. dos Santos Carvalho A. Bohn L. Ramos N. C. Abdalla P. P. (2022). Nutritional status, health risk behaviors, and eating habits are correlated with physical activity and exercise of Brazilian older hypertensive adults: a cross-sectional study. BMC Public Health 22:2382. doi: 10.1186/s12889-022-14873-4, PMID: 36536331 PMC9762644

[ref18] Dakanalis A. Mentzelou M. Papadopoulou S. Papandreou D. Spanoudaki M. Vasios G. K. . (2023). The association of emotional eating with overweight/obesity, depression, anxiety/stress, and dietary patterns: a review of the current clinical evidence. Nutrients 15:1173. doi: 10.3390/nu15051173, PMID: 36904172 PMC10005347

[ref19] Dent E. Wright O. R. L. Woo J. Hoogendijk E. (2023). Malnutrition in older adults. Lancet 401, 951–966. doi: 10.1016/S0140-6736(22)02612-5, PMID: 36716756

[ref20] Dominguez L. Veronese N. Baiamonte E. Guarrera M. Parisi A. Ruffolo C. . (2022). Healthy aging and dietary patterns. Nutrients 14:889. doi: 10.3390/nu14040889, PMID: 35215539 PMC8879056

[ref21] Donges U.-S. Suslow T. (2017). Alexithymia and automatic processing of emotional stimuli: a systematic review. Rev. Neurosci. 28, 247–264. doi: 10.1515/revneuro-2016-0049, PMID: 28099136

[ref22] Dubois L. Bédard B. Goulet D. Prud’homme D. Tremblay R. E. Boivin M. (2022). Eating behaviors, dietary patterns and weight status in emerging adulthood and longitudinal associations with eating behaviors in early childhood. Int. J. Behav. Nutr. Phys. Act. 19:139. doi: 10.1186/s12966-022-01376-z, PMID: 36384744 PMC9670577

[ref23] Fadnes L. Økland J.-M. Haaland Ø. Johansson K. (2022). Estimating impact of food choices on life expectancy: a modeling study. PLoS Med. 19:e1003889. doi: 10.1371/journal.pmed.1003889, PMID: 35134067 PMC8824353

[ref24] Fantini-Hauwel C. Gois C. Luminet O. Banse E. Bigot A. Noel X. . (2025). Exploring alexithymia with the French Perth alexithymia questionnaire: latent structure, profiles, and links with affective outcomes. Front. Psychol. 16:1615612. doi: 10.3389/fpsyg.2025.1615612, PMID: 40599529 PMC12209315

[ref25] Favieri F. (2021). Emotional eating, alexithymia and weight gain in healthy young adults. Mediterranean J. Clin. Psychol. 9, 10–11.

[ref26] Fernández R. S. Crivelli L. Guimet N. M. Allegri R. F. Pedreira M. E. (2020). Psychological distress associated with COVID-19 quarantine: latent profile analysis, outcome prediction and mediation analysis. J. Affect. Disord. 277, 75–84. doi: 10.1016/j.jad.2020.07.133, PMID: 32799107 PMC7413121

[ref27] Gaggero G. Bizzego A. Dellantonio S. Pastore L. Lim M. Esposito G. (2021). Clarifying the relationship between alexithymia and subjective interoception. PLoS One 16:e0261126. doi: 10.1371/journal.pone.0261126, PMID: 34898643 PMC8668127

[ref28] Ge P. Wang X. Gao S. Liu J. Niu Y. Yan M. . (2023). Reliability and validity of the Chinese version of the Sakata eating behavior scale short form and preliminary analysis of the factors related to the score of the scale. Front. Nutr. 10:1076209. doi: 10.3389/fnut.2023.1076209, PMID: 36969818 PMC10031001

[ref29] Gerstberger L. Blanke E. S. Keller J. Brose A. (2023). Stress buffering after physical activity engagement: an experience sampling study. Br. J. Health Psychol. 28, 876–892. doi: 10.1111/bjhp.12659, PMID: 37037566

[ref30] Godet A. Fortier A. Bannier E. Coquery N. Val-Laillet D. (2022). Interactions between emotions and eating behaviors: main issues, neuroimaging contributions, and innovative preventive or corrective strategies. Rev. Endocr. Metab. Disord. 23, 807–831. doi: 10.1007/s11154-021-09700-x, PMID: 34984602

[ref31] Godos J. Currenti W. Angelino D. Mena P. Castellano S. Caraci F. . (2020). Diet and mental health: review of the recent updates on molecular mechanisms. Antioxidants 9:346. doi: 10.3390/antiox9040346, PMID: 32340112 PMC7222344

[ref32] Goetz D. B. Johnson E. C. Naugle A. E. Borges L. M. (2020). Alexithymia, state-emotion dysregulation, and eating disorder symptoms: a mediation model. Clin. Psychol. 24, 166–175. doi: 10.1111/cp.12210

[ref33] Guidotti S. Torelli P. Ambiveri G. Fiduccia A. Chirco D. Pruneti C. (2025). Mediation analysis of anxiety and depression between alexithymia and frequency of headache attacks and impact on suicidal ideation in primary headache. J. Affect. Disord. 388:119716. doi: 10.1016/j.jad.2025.119716, PMID: 40555347

[ref34] Hao M. Fang Y. Yan W. Gu J. Hao Y. Wu C. (2022). Relationship between body dissatisfaction, insufficient physical activity, and disordered eating behaviors among university students in southern China. BMC Public Health 22:2054. doi: 10.1186/s12889-022-14515-9, PMID: 36352371 PMC9648036

[ref35] Pink A. E. Lee M. Price M. Williams C. (2019). A serial mediation model of the relationship between alexithymia and BMI: The role of negative affect, negative urgency and emotional eating. Appetite. 133, 270–278. doi: 10.1016/j.appet.2018.11.014 30465802

[ref36] Hawash M. Alhazmi A. El-Sayed M. Mushfiq S. El-Ashry A. Ahmed H. A. . (2023). Emotional eating behaviors in later life: identifying key factors for healthy aging. Geriatr. Nurs. 55, 152–160. doi: 10.1016/j.gerinurse.2023.11.012 37995607

[ref37] Hill D. Conner M. Clancy F. Moss R. H. Wilding S. Bristow M. . (2021). Stress and eating behaviours in healthy adults: a systematic review and meta-analysis. Health Psychol. Rev. 16, 280–304. doi: 10.1080/17437199.2021.1923406 33913377

[ref38] Hill J. Meredith P. Forrester G. Shirley J. Gomersall S. R. (2024). Understanding the relationship between attachment orientation and physical activity participation: an exploratory study. J. Phys. Act. Health 21, 1019–1026. doi: 10.1123/jpah.2023-0717, PMID: 39025468

[ref39] Hong W. Bernacki M. L. Perera H. N. (2020). A latent profile analysis of undergraduates’ achievement motivations and metacognitive behaviors, and their relations to achievement in science. J. Educ. Psychol. 112, 1409–1430. doi: 10.1037/edu0000445

[ref40] Hou C. Shi H. Ma Y. Chou J. (2025). Heterogeneity of alexithymia subgroups: a factor mixture modelling approach. Eur. J. Psychol. Assess. 41, 4–12. doi: 10.1027/1015-5759/a000828

[ref41] Hu C. Bin J. Zhang W. Huang Y. (2025). How sports-implied packaging of protein powder products enhances the purchase intention of generation Z: evidence from multiple experiments. Front. Nutr. 12:1645614. doi: 10.3389/fnut.2025.1645614 41323995 PMC12661654

[ref42] Hu C. Huang Y. Zhang W. (2025). Childhood emotional abuse and suicidal ideation in college students: exploring the mediating role of alexithymia and the moderating effect of physical exercise. Front. Psychiatry 16:1660164. doi: 10.3389/fpsyt.2025.1660164 PMC12698450 41393228

[ref43] Hu C. Zhang W. Huang W. Jin C. (2025). How grit enhances physical exercise in college students: mediating roles of personal growth initiative and self-efficacy. Front. Psychol. 16:1652984. doi: 10.3389/fpsyg.2025.1652984, PMID: 40994858 PMC12455857

[ref44] Huang W. Chen B. Hu C. (2025a). The latent profile structure of negative emotion in female college students and its impact on eating behavior: the mediating role of physical exercise. Front. Public Health 13:1663474. doi: 10.3389/fpubh.2025.1663474, PMID: 40880924 PMC12380551

[ref45] Huang W. Chen B. Hu C. (2025b). Exploring self-rated health, physical activity, and social anxiety among female Chinese university students: a variable- and person-centered analysis. Front. Public Health 13:1681504. doi: 10.3389/fpubh.2025.1681504, PMID: 41048258 PMC12488654

[ref46] Kärkkäinen U. Mustelin L. Raevuori A. Kaprio J. Keski-Rahkonen A. (2018). Do disordered eating behaviours have long-term health-related consequences? Eur. Eat. Disord. Rev. 26, 22–28. doi: 10.1002/erv.2568, PMID: 29160017 PMC5732059

[ref47] Keech J. J. Hamilton K. (2022). An integrated dual-process model for coping behaviour. Stress. Health 38, 591–601. doi: 10.1002/smi.3121, PMID: 34921495

[ref48] Kim E. Lee Y. Shin J. Kim G. Yoon J. (2021). Effects of health-promoting lifestyles in midlife on cognitive functioning in later life. Innov. Aging 5:309. doi: 10.1093/geroni/igab046.1197, PMID: 40799322

[ref49] Krasnow I. D. Smith T. Linnan L. Mclellan D. Hino S. Hunter J. C. (2014). Can we say what diet is best for health? Annu. Rev. Public Health 35, 83–103. doi: 10.1146/annurev-publhealth-032013-182351 24641555

[ref50] Kvalsvik F. Øgaard T. Jensen Ø. (2021). Environmental factors that impact the eating behavior of home-living older adults. Int. J. Nurs. Stud. Adv. 3:100046. doi: 10.1016/j.ijnsa.2021.100046, PMID: 38746717 PMC11080564

[ref51] Lanfredi M. Macis A. Ferrari C. Meloni S. Pedrini L. Ridolfi M. E. . (2021). Maladaptive behaviours in adolescence and their associations with personality traits, emotion dysregulation and other clinical features in a sample of Italian students: a cross-sectional study. Borderline Personal. Disord. Emot. Dysregul. 8:14. doi: 10.1186/s40479-021-00154-w, PMID: 33941285 PMC8094601

[ref52] Levinsky M. Schiff M. (2021). Lifetime cumulative adversity and physical health deterioration in old age: evidence from a fourteen-year longitudinal study. Soc. Sci. Med. 289:114407. doi: 10.1016/j.socscimed.2021.114407, PMID: 34555682

[ref53] Li L. Wang P. Li S. Liu Q. Yu F. Guo Z. . (2023). Canonical correlation analysis of depression and anxiety symptoms among college students and their relationship with physical activity. Sci. Rep. 13:11516. doi: 10.1038/s41598-023-38682-w, PMID: 37460562 PMC10352328

[ref54] Liang D. (1994). Stress levels Among college students and their relationship with physical exercise. Chin. Ment. Health J. 8(1):2.

[ref55] Longo V. Anderson R. M. (2022). Nutrition, longevity and disease: from molecular mechanisms to interventions. Cell 185, 1455–1470. doi: 10.1016/j.cell.2022.04.002, PMID: 35487190 PMC9089818

[ref56] Lui P. P. (2015). Intergenerational cultural conflict, mental health, and educational outcomes among Asian and Latino/a Americans: qualitative and meta-analytic review. Psychol. Bull. 141, 404–446. doi: 10.1037/a0038449, PMID: 25528344

[ref57] LuminetO. NielsonK. A. (2025). Alexithymia: toward an experimental, processual affective science with effective interventions. Annu. Rev. Psychol. 76:741. doi: 10.1146/annurev-psych-021424-030718, PMID: 39322432

[ref58] Lundh L.-G. Simonsson-Sarnecki M. (2001). Alexithymia, emotion, and somatic complaints. J. Pers. 69, 483–510. doi: 10.1111/1467-6494.00153, PMID: 11478734

[ref59] Lyvers M. Kelahroodi M. Udodzik E. Stapleton P. Thorberg F. A. (2022). Alexithymia and binge eating: maladaptive emotion regulation strategy or deficient interoception? Appetite 175:106073. doi: 10.1016/j.appet.2022.106073, PMID: 35568089

[ref60] Marx W. Lane M. Hockey M. Aslam H. Berk M. Walder K. . (2020). Diet and depression: exploring the biological mechanisms of action. Mol. Psychiatry 26, 134–150. doi: 10.1038/s41380-020-00925-x, PMID: 33144709

[ref61] McAtamney K. Mantzios M. Egan H. Wallis D. J. (2023). A systematic review of the relationship between alexithymia and emotional eating in adults. Appetite 180:106279. doi: 10.1016/j.appet.2022.106279, PMID: 36087827

[ref62] Montana J. I. Matamala-Gomez M. Maisto M. Mavrodiev P. A. Cavalera C. M. Diana B. . (2020). The benefits of emotion regulation interventions in virtual reality for the improvement of wellbeing in adults and older adults: a systematic review. J. Clin. Med. 9:500. doi: 10.3390/jcm9020500, PMID: 32059514 PMC7073752

[ref63] Muir X. Preece D. A. Becerra R. (2024). Alexithymia and eating disorder symptoms: the mediating role of emotion regulation. Aust. Psychol. 59, 121–131. doi: 10.1080/00050067.2023.2236280

[ref64] Neumann D. Hammond F. M. Sander A. M. Bogner J. Bushnik T. Finn J. A. . (2025). Alexithymia prevalence, characterization, and associations with emotional functioning and life satisfaction: a traumatic brain injury model system study. J. Head Trauma Rehabil. 40:E175. doi: 10.1097/HTR.0000000000000967, PMID: 39146446

[ref65] Ni Y. Fang S. (2025). Does the Chinese version of 20-item Toronto alexithymia scale (TAS-20-C) measure alexithymia in Chinese young adolescents? Evidence from confirmatory factor analysis, network analysis, and latent profile analysis. BMC Psychol. 13:687. doi: 10.1186/s40359-025-03028-w, PMID: 40597410 PMC12220322

[ref66] Norman K. Haß U. Pirlich M. (2021). Malnutrition in older adults—recent advances and remaining challenges. Nutrients 13:2764. doi: 10.3390/nu13082764, PMID: 34444924 PMC8399049

[ref67] Obeid S. Hallit S. Akel M. Brytek-Matera A. (2021). Orthorexia nervosa and its association with alexithymia, emotion dysregulation and disordered eating attitudes among Lebanese adults. Eat. Weight Disord. 26, 2607–2616. doi: 10.1007/s40519-021-01112-9, PMID: 33570743 PMC7877311

[ref68] Perchtold-Stefan C. M. Fink A. Rominger C. Weiss E. M. Papousek I. (2020). More habitual physical activity is linked to the use of specific, more adaptive cognitive reappraisal strategies in dealing with stressful events. Stress. Health 36, 274–286. doi: 10.1002/smi.2929, PMID: 31957957 PMC7497133

[ref69] Poggiogalle E. Kiesswetter E. Romano M. Saba A. Sinesio F. Polito A. . (2020). Psychosocial and cultural determinants of dietary intake in community-dwelling older adults: a determinants of diet and physical activity systematic literature review. Nutrition 85, 337–341. doi: 10.1016/j.nut.2020.111131 33545539

[ref70] Preece D. Gross J. J. (2023). Conceptualizing alexithymia. Pers. Individ. Differ. 215:112375. doi: 10.1016/j.paid.2023.112375

[ref71] Preece D. Mehta A. Becerra R. Chen W. Allan A. Robinson K. . (2021). Why is alexithymia a risk factor for affective disorder symptoms? The role of emotion regulation. J. Affect. Disord. 296, 337–341. doi: 10.1016/j.jad.2021.09.085 34606815

[ref72] Preece D. A. Mehta A. Petrova K. Sikka P. Bjureberg J. Becerra R. . (2023). Alexithymia and emotion regulation. J. Affect. Disord. 324, 232–238. doi: 10.1016/j.jad.2022.12.065 36566943

[ref73] Proença Lopes C. Allado E. Poussel M. Hamroun A. Essadek A. Albuisson E. . (2022). An association between alexithymia and the characteristics of sport practice: a multicenter, cross-sectional study. Healthcare 10:432. doi: 10.3390/healthcare10030432, PMID: 35326910 PMC8950812

[ref74] Qiu C. Hou M. (2020). Association between food preferences, eating behaviors and socio-demographic factors, physical activity among children and adolescents: a cross-sectional study. Nutrients 12:640. doi: 10.3390/nu12030640, PMID: 32121152 PMC7146169

[ref75] Quinto R. M. Russo F. Scafuto F. Innamorati M. Montecucco F. N. Ghiroldi S. (2025). Effects of a body-based mindfulness program on alexithymia, dispositional mindfulness, and distress symptoms: a pilot clinical trial. Behav. Sci. 15:55. doi: 10.3390/bs15010055, PMID: 39851859 PMC11763314

[ref76] Robinson E. Boyland E. Chisholm A. Harrold J. Maloney N. G. Marty L. . (2021). Obesity, eating behavior and physical activity during COVID-19 lockdown: a study of UK adults. Appetite 156:104853. doi: 10.1016/j.appet.2020.104853, PMID: 33038479 PMC7540284

[ref77] Rook K. S. Charles S. T. (2017). Close social ties and health in later life: strengths and vulnerabilities. Am. Psychol. 72, 567–577. doi: 10.1037/amp0000104, PMID: 28880103 PMC5599123

[ref78] Rossi A. A. (2025). Tying food addiction to uncontrolled eating: the roles of eating-related thoughts and emotional eating. Nutrients 17:369. doi: 10.3390/nu17030369, PMID: 39940227 PMC11819927

[ref79] Shizuma H. Abe T. Kanbara K. Amaya Y. Mizuno Y. Saka-Kochi Y. . (2021). Interoception and alexithymia are related to differences between the self-reported and the objectively measured physical activity in patients with chronic musculoskeletal pain. J. Psychosom. Res. 140:110324. doi: 10.1016/j.jpsychores.2020.110324, PMID: 33278660

[ref80] Simone M. Telke S. Anderson L. M. Eisenberg M. Neumark-Sztainer D. (2022). Ethnic/racial and gender differences in disordered eating behavior prevalence trajectories among women and men from adolescence into adulthood. Soc. Sci. Med. 294:114720. doi: 10.1016/j.socscimed.2022.114720, PMID: 35033795 PMC8821169

[ref81] Spencer S. Korosi A. Layé S. Shukitt-Hale B. Barrientos R. (2017). Food for thought: how nutrition impacts cognition and emotion. NPJ Sci. Food. 1:7. doi: 10.1038/s41538-017-0008-y, PMID: 31304249 PMC6550267

[ref82] Spurk D. Hirschi A. Wang M. Valero D. Kauffeld S. (2020). Latent profile analysis: a review and “how to” guide of its application within vocational behavior research. J. Vocat. Behav. 120:103445. doi: 10.1016/j.jvb.2020.103445

[ref83] Strodl E. Wylie L. (2020). Childhood trauma and disordered eating: exploring the role of alexithymia and beliefs about emotions. Appetite 154:104802. doi: 10.1016/j.appet.2020.104802, PMID: 32717292

[ref84] Tayama J. Ogawa S. Takeoka A. Kobayashi M. Shirabe S. (2017). Item response theory-based validation of a short form of the eating behavior scale for Japanese adults. Medicine 96:e8334. doi: 10.1097/MD.0000000000008334, PMID: 29049248 PMC5662414

[ref85] Teh W. L. Abdin E. PV A. Siva Kumar F. D. Roystonn K. Wang P. . (2023). Measuring social desirability bias in a multi-ethnic cohort sample: its relationship with self-reported physical activity, dietary habits, and factor structure. BMC Public Health 23:415. doi: 10.1186/s12889-023-15309-3, PMID: 36859251 PMC9979418

[ref86] Teixeira P. J. Carraça E. V. Markland D. Silva M. N. Ryan R. M. (2012). Exercise, physical activity, and self-determination theory: a systematic review. Int. J. Behav. Nutr. Phys. Act. 9, 78–30. doi: 10.1186/1479-5868-9-78, PMID: 22726453 PMC3441783

[ref87] Tella M. D. Adenzato M. Catmur C. Miti F. Castelli L. Ardito R. (2020). The role of alexithymia in social cognition: evidence from a non-clinical population. J. Affect. Disord. 273, 482–492. doi: 10.1016/j.jad.2020.05.012 32560944

[ref88] Turton R. Chami R. Treasure J. (2017). Emotional eating, binge eating and animal models of binge-type eating disorders. Curr. Obes. Rep. 6, 217–228. doi: 10.1007/s13679-017-0265-8, PMID: 28434108

[ref89] Varela C. Andrés A. Saldaña C. (2020). The behavioral pathway model to overweight and obesity: coping strategies, eating behaviors and body mass index. Eat. Weight Disord. 25, 1277–1283. doi: 10.1007/s40519-019-00760-2, PMID: 31376111

[ref90] Vuillier L. Carter Z. Teixeira A. R. Moseley R. L. (2020). Alexithymia may explain the relationship between autistic traits and eating disorder psychopathology. Mol. Autism. 11:63. doi: 10.1186/s13229-020-00364-z, PMID: 32758290 PMC7406391

[ref91] Walker-Clarke A. Walasek L. Meyer C. (2022). Psychosocial factors influencing the eating behaviours of older adults: a systematic review. Ageing Res. Rev. 77:101597. doi: 10.1016/j.arr.2022.101597, PMID: 35219902

[ref92] Wang X. Wu Y. Miao J. Pu K. Ming W. Zang S. (2023). Factors associated with eating behaviors in older adults from a socioecological model perspective. BMC Public Health 23:1726. doi: 10.1186/s12889-023-16651-2, PMID: 37670266 PMC10481492

[ref93] Wang K. Yang Y. Zhang T. Ouyang Y. Liu B. Luo J. (2020). The relationship between physical activity and emotional intelligence in college students: the mediating role of self-efficacy. Front. Psychol. 11:967. doi: 10.3389/fpsyg.2020.00967, PMID: 32581908 PMC7296084

[ref94] Wen J. Xu Q. Jiang Y. Li M. (2024). The effects of student bullying on non-suicidal self-injurious behavior in rural adolescents: the chain-mediated effects of alexithymia and ruminate thinking. Front. Psychol. 15:1483408. doi: 10.3389/fpsyg.2024.1483408, PMID: 39737230 PMC11684095

[ref95] Wheeler K. Greiner P. Boulton M. (2005). Exploring alexithymia, depression, and binge eating in self-reported eating disorders in women. Perspect. Psychiatr. Care 41, 114–123. doi: 10.1111/j.1744-6163.2005.00022.x, PMID: 16138820

[ref96] White R. L. Vella S. Biddle S. Sutcliffe J. Guagliano J. M. Uddin R. . (2024). Physical activity and mental health: a systematic review and best-evidence synthesis of mediation and moderation studies. Int. J. Behav. Nutr. Phys. Act. 21:134. doi: 10.1186/s12966-024-01676-6, PMID: 39609855 PMC11603721

[ref97] Wu Q. Gao Z. Yu X. Wang P. (2022). Dietary regulation in health and disease. Signal Transduct. Target. Ther. 7:252. doi: 10.1038/s41392-022-01104-w, PMID: 35871218 PMC9308782

[ref98] Wu R. Jing L. Liu Y. Wang H. Yang J. (2022). Effects of physical activity on regulatory emotional self-efficacy, resilience, and emotional intelligence of nurses during the COVID-19 pandemic. Front. Psychol. 13:1059786. doi: 10.3389/fpsyg.2022.1059786, PMID: 36571052 PMC9780437

[ref99] Xu J. Shang B. Zhang J. Luo C. Bian Z. Lv F. (2024). The effect of alexithymia on self-perceived aging among community-dwelling older adults with multiple chronic conditions: the mediating role of maladaptive cognitive emotion regulation strategies. Front. Psych. 15:1437478. doi: 10.3389/fpsyt.2024.1437478, PMID: 39583754 PMC11582025

[ref100] Yang X. Wang M. Wang J. Zhang S. Yang X. Zhao L. (2024). Physical literacy and health of Chinese medical students: the chain mediating role of physical activity and subjective well-being. Front. Public Health 12:1348743. doi: 10.3389/fpubh.2024.1348743, PMID: 39056080 PMC11269216

[ref101] Yurtdaş Depboylu G. Fındık B. E. (2024). Relationships among alexithymia, psychological distress, and disordered eating behaviors in adolescents. Appetite 200:107536. doi: 10.1016/j.appet.2024.107536, PMID: 38825016

[ref102] Zhang G. Feng W. Zhao L. Zhao X. Li T. (2024). The association between physical activity, self-efficacy, stress self-management and mental health among adolescents. Sci. Rep. 14:5488. doi: 10.1038/s41598-024-56149-4, PMID: 38448518 PMC10917799

[ref103] Zhang R. Zhang M. Wang P. (2025). The intricate interplay between dietary habits and cognitive function: insights from the gut-brain axis. Front. Nutr. 12:1539355. doi: 10.3389/fnut.2025.1539355, PMID: 39944956 PMC11813792

[ref104] Zheng J. Zhou R. Li F.-R. Chen L. Wu K. Huang J. . (2021). Association between dietary diversity and cognitive impairment among the oldest-old: findings from a nationwide cohort study. Clin. Nutr. 40, 1452–1462. doi: 10.1016/j.clnu.2021.02.041, PMID: 33740515

